# The Relationship between Sleep-Wake Cycle and Cognitive Functioning in Young People with Affective Disorders

**DOI:** 10.1371/journal.pone.0124710

**Published:** 2015-04-21

**Authors:** Joanne S. Carpenter, Rébecca Robillard, Rico S. C. Lee, Daniel F. Hermens, Sharon L. Naismith, Django White, Bradley Whitwell, Elizabeth M. Scott, Ian B. Hickie

**Affiliations:** Clinical Research Unit, Brain & Mind Research Institute, University of Sydney, Camperdown, NSW, Australia

## Abstract

Although early-stage affective disorders are associated with both cognitive dysfunction and sleep-wake disruptions, relationships between these factors have not been specifically examined in young adults. Sleep and circadian rhythm disturbances in those with affective disorders are considerably heterogeneous, and may not relate to cognitive dysfunction in a simple linear fashion. This study aimed to characterise profiles of sleep and circadian disturbance in young people with affective disorders and examine associations between these profiles and cognitive performance. Actigraphy monitoring was completed in 152 young people (16–30 years; 66% female) with primary diagnoses of affective disorders, and 69 healthy controls (18–30 years; 57% female). Patients also underwent detailed neuropsychological assessment. Actigraphy data were processed to estimate both sleep and circadian parameters. Overall neuropsychological performance in patients was poor on tasks relating to mental flexibility and visual memory. Two hierarchical cluster analyses identified three distinct patient groups based on sleep variables and three based on circadian variables. Sleep clusters included a ‘long sleep’ cluster, a ‘disrupted sleep’ cluster, and a ‘delayed and disrupted sleep’ cluster. Circadian clusters included a ‘strong circadian’ cluster, a ‘weak circadian’ cluster, and a ‘delayed circadian’ cluster. Medication use differed between clusters. The ‘long sleep’ cluster displayed significantly worse visual memory performance compared to the ‘disrupted sleep’ cluster. No other cognitive functions differed between clusters. These results highlight the heterogeneity of sleep and circadian profiles in young people with affective disorders, and provide preliminary evidence in support of a relationship between sleep and visual memory, which may be mediated by use of antipsychotic medication. These findings have implications for the personalisation of treatments and improvement of functioning in young adults early in the course of affective illness.

## Introduction

Adolescence and young adulthood is a key developmental period with a heightened incidence of affective disorders (defined in this paper to include major depressive disorder, bipolar disorder, and anxiety disorders) [1, 2]. Affective disorders have been associated with a range of cognitive deficits, including dysfunction in areas of new learning, memory, attention, and executive functioning [3–8]. Many of these deficits are present in the first episode of mental illness [9, 10] and have been shown to persist into remission [11–13]. This may be reflective of such deficits being risk-factors for the development or recurrence of these disorders, or epiphenomena of other causal factors or risk-factors. Poorer neuropsychological performance has been linked to poorer clinical, social, and occupational outcomes [14–16] and as such uncovering any potential causal or mediational factors is important to identify targets for therapeutic interventions.

One factor that could potentially be associated with cognitive dysfunctions in affective disorders is sleep and circadian rhythm disturbance. Sleep disturbances are found in major depressive disorder (difficulty initiating and maintaining sleep, and abnormal sleep duration [17, 18]), anxiety disorders (altered sleep duration, insomnia [18–21]), and bipolar disorder (decreased sleep duration during manic and hypomanic episodes [22, 23]). Sleep disturbances in affective disorders often persist into remission [18, 24–26], and are associated with greater illness severity [27], poorer treatment response [28, 29], and greater risk of relapse [30, 31]. Abnormal patterns in the 24hour rhythm of rest and activity are also found in those with affective disorders including a delay in the phase of sleep-wake timing [32–34], less regular patterns of daily activity [35], and a lower amplitude of the circadian rest-activity cycle [36, 37]. Measurement of endogenous circadian rhythms in those with affective disorders suggests a biological component to these abnormalities, with reports of irregular patterns of hormone secretion, temperature regulation, and gene expression [38–42]. This biological link could be reflective of an underlying causal pathway to affective dysfunction and thus has the potential to explain other dysfunctions associated with risk or development of affective disorders, such as cognitive deficits. Further support for a causal role of sleep and circadian disturbance comes from evidence suggesting that sleep disturbance, later sleep-wake phases, and more variable rest and activity rhythms may be premorbid risk factors or early indicators of emerging affective disorders [43–45]. The link between cognition and sleep-wake factors is well supported in research in psychiatrically healthy individuals. Sleep restriction [46–48] and insomnia [49], as well as poor sleep quality and shorter sleep duration [50–52], have all been associated with poorer cognitive performance. There is also evidence of an influence of circadian disruption on cognition, with irregular bedtimes in children [53], more fragmented rest and activity patterns in older adults [54, 55], and circadian disruption as caused by shift-work associated with poorer cognitive performance [56]. In addition, cognitive performance itself follows a circadian rhythm [57, 58], and thus it follows that circadian disruption may affect cognitive function.

There is some evidence that sleep-wake factors may play a role in the cognitive dysfunction found in affective disorders. This includes reports in older samples of poorer cognitive performance in those with poorer subjective sleep quality and subclinical depressive symptoms [59], and clinician-rated insomnia in moderate to severe major depression [60], and increased nocturnal awakenings in those with largely remitted depressive symptoms [61]. In adults with major depressive disorder, initial evidence suggests that poorer visual memory may be associated with shorter total sleep time and a lower amount of rapid eye movement (REM) sleep [62], and verbal memory retention may be linked to higher amounts of slow wave sleep (SWS) [63]. We have reported previously that circadian disruption (as measured by melatonin secretion) is related to verbal memory performance in young people with affective disorders [64]. Aside from this, there has been little consideration of the potential of sleep and circadian variables to influence cognition in youth with affective disorders.

Although sleep and circadian disturbances are widely reported in affective disorders, there exists a considerable degree of heterogeneity [65]. For example abnormalities in sleep duration include both hypo- and hypersomnia [18, 66], and there are contrasting reports of both advanced and delayed endogenous circadian phase [38, 39, 42, 67, 68]. This may be indicative of different underlying phenotypes, which may be differentially related to symptoms, severity, and functional correlates of affective disorders. These underlying phenotypes may not be related to a specific diagnosis: evidence suggests that sleep disturbance is a transdiagnostic factor across both affective and psychotic disorders [69–71]. Further, traditional diagnostic categories may not be the most useful way in which to group patients in terms of aetiology, risk factors, or treatment response [72], and thus the utility of classifications based on other factors are of considerable interest.

The current study aimed to investigate the relationship between patterns of sleep and circadian function (as measured by actigraphy) and neuropsychological performance in young people with affective disorders. Given the high degree of heterogeneity and potentially distinct phenotypes of sleep and circadian rhythm disturbances, we used cluster analysis to determine whether distinct sleep or circadian profiles were related to cognition in these young adults. Cluster analysis is an analytic technique for identifying subgroups of patients based on their similarities across a particular set of variables. This data-driven method allows identification of underlying structure without imposing pre-conceived notions regarding other variables of interest, such as diagnosis. It was hypothesised that profiles defined by greater sleep disturbances, more delayed sleep timing, and less regular circadian rhythms would be associated with poorer cognitive performance.

## Materials and Methods

### Participants

One hundred and fifty-two outpatients (100 females) aged 16 to 30 years were recruited from specialized referral services for the assessment and early intervention of mental health problems in young people [73, 74]. Primary diagnoses were major depressive disorder (n = 87), bipolar disorder (n = 46; 11 bipolar I, 27 bipolar II, 8 bipolar not otherwise specified), or anxiety disorder (n = 19; 1 panic disorder, 4 obsessive compulsive disorder, 6 generalized anxiety disorder, 8 social anxiety disorder) as established by a mental health professional according to DSM-IV-TR criteria [75]. For those patients who were receiving psychotropic medication, assessment was under ‘treatment as usual’ conditions: medications were not interrupted in any way. Medication information was missing for 14 patients (9.2%). For the remaining participants, at the time of assessment 27% of patients were not taking any psychotropic medications, and 73% were taking at least one type of medication (antidepressants-46%, antipsychotics-30%, mood stabilisers-24%, sedatives or hypnotics-9%, melatonin or agomelatine-11%, other-2%).

In addition, sixty nine healthy controls (39 females) aged 18 to 30 years, were recruited from the community in the same metropolitan area as the outpatients and completed the actigraphy monitoring component of the study.

Exclusion criteria for all participants were history of neurological disease (e.g. epilepsy), medical illness known to impact cognitive and brain function (e.g. cancer), electroconvulsive therapy in the last 3 months, intellectual and/or developmental disability, insufficient English language skills, and current substance dependence.

### Ethics Statement

The study was approved by the University of Sydney Human Research Ethics Committee and all participants gave written informed consent. In accordance with Australian law and ethics committee guidelines, additional parental consent was not required as all participants were aged 16 years and over.

### Clinical and Neuropsychological Assessment

For all outpatient participants, a clinical assessment was conducted by a psychiatrist or trained research psychologist, and the research psychologist also conducted a neuropsychological assessment. The clinical assessments consisted of a semi-structured interview [16], which included the Hamilton Depression Rating Scale (HDRS, 17-item: [76]) and the Social and Occupational Functioning Assessment Scale (SOFAS: [77]). HDRS total scores were calculated without including the three insomnia items in order measure depressive symptomology as distinct from sleep disturbances.

A comprehensive battery of neuropsychological tests was administered on the same day as the clinical assessment. All participants were asked to abstain from drug or alcohol use for 48 hours before testing and informed that they may be asked to undertake an alcohol breath test and/or a saliva drug screen. The Wechsler Test of Adult Reading was used to estimate intelligence quotient [78]. The following measures were taken from the battery to be included in analysis: Trail-Making Test parts A and B completion time (TMT-A and TMT-B: [79]), Rey-Osterrieth Complex Figure Test- 3 minute delay score (RCFT: [79]), Rey Auditory Verbal Learning Test (RAVLT [79])- immediate recall (sum of trials 1–5), and 20 min delayed recall (trial 7), total words on the letters (FAS) subtest of the Controlled Oral Word Association Test (COWAT: [79]), the Cambridge Automated Neuropsychological Testing Battery (CANTAB: [80]) average five-choice Reaction Time, Intra/Extra Dimensional Shift total adjusted errors, Rapid Visual Information Processing task- A prime (sensitivity to the target), Paired Associate Learning total adjusted errors, and Spatial Span test maximum span length. Neuropsychological variables were converted to “demographically corrected” standardized scores based on established norms: TMT [81], ROCF [82], RAVLT [83], COWAT [81], and CANTAB variables (internal normative database of the 3000 healthy volunteers; http://www.cantab.com). Outliers beyond ±3.0 z-scores were curtailed to +3.0 or -3.0 (depending on the direction) to minimise the effects of skewed distributions. The number of cases beyond -3.0 did not exceed 10% for any variables and there were no cases beyond +3.0.

### Actigraphy

All participants filled out a sleep diary and wore a wrist actiwatch on their non-dominant arm for approximately 14 days (Actiwatch- 64, Actiwatch-L, Actiwatch-2 or Actiwatch Spectrum: Philips Respironics, OR; number of days with valid data ranged from 5 to 22). The majority of participants from the outpatient group (90%, n = 137) completed actigraphy within 30 days of their neuropsychological and clinical assessments. The remaining 10% (n = 15) completed their actigraphy within 100 days of their neuropsychological and clinical assessments (mean time gap±SD: 12 days±20). Actigraphy data was sampled at a 1-minute acquisition rate for Actiwatch-Ls and a 30-second rate for all other models. Estimates of sleep onset and offset times were automatically determined with Actiware 5.0 software (Minimitter-Respironics Inc., Bend, OR, USA), and confirmed or adjusted by qualified technicians using visual inspection and the sleep diaries. This method provides an indirect estimate of sleep, however the terms ‘sleep’ and ‘wake’ are used here for descriptive purposes. The same software was used to identify periods of wake during the sleep episode with a medium sensitivity threshold of 40 counts per epoch. To characterise the 24-hour rest-activity cycle, individual activity counts were log transformed and fitted to an extended cosinor model [84] using non-linear least squares regression (GraphPad Software 6.0, San Diego California USA). The following sleep and circadian parameters were generated: average sleep onset and offset times (timing of the onset and offset of the rest episode), average wake after sleep onset (WASO: the duration of awakenings during the rest period), average total sleep time (TST: total time between sleep onset and sleep offset times), average sleep midpoint (time point halfway between sleep onset and sleep offset), cosinor acrophase (time point at which the best-fit curve of the rest-activity cycle reaches its peak), amplitude (range of activity levels), and R^2^ (goodness of fit measure considered to reflect the strength of circadian rhythmicity). Due to equipment error and data loss, cosinor analyses were unable to be completed for nine patients and six controls. Outlying values on any sleep or circadian variable (9 values) were removed.

### Statistical Analysis

The Statistical Package for Social Sciences (SPSS for Windows 22.0: SPSS, Inc., Chicago, IL, USA) was used for the following analyses. A p value of. 05 was used for all comparisons unless otherwise stated.

T-tests and Chi-squared tests were used to compare patients to controls on age and gender distributions. One-way analysis of variance (ANOVA) or analysis of covariance (ANCOVA- controlling for significant age or gender differences) was used to compare patients to controls across sleep and circadian variables. Correlations were used to examine relationships between neuropsychological variables and sleep and circadian variables within the patient group. Given the large number of correlations a p value of. 01 was used to reduce the risk of type I error.

To identify patterns of sleep and circadian rhythms in the patient group, we performed two hierarchical cluster analyses: the first utilised only the sleep variables (sleep onset time, sleep offset time, total sleep time, and WASO) and the second only the circadian variables (sleep midpoint, acrophase, amplitude and R^2^). In order to have comparable variables within each cluster analysis, values across sleep and circadian variables were transformed into standardized z-scores. Ward’s method [85] was used as the linkage measure and squared Euclidean distance was used as the similarity measure. This agglomerative hierarchical method was used as it does not impose preconceived notions regarding the number of clusters and is thought to be effective at uncovering underlying structure [86]. The change in agglomeration coefficients was used to determine a demarcation point, and this, in combination with visual inspection of the dendrogram, confirmed the optimal number of clusters. One-way ANOVA with Bonferroni-corrected pairwise comparisons was then used to characterise these groups in comparison to the control group across the sleep and circadian clustering variables. The control group was not included in the cluster analysis as the aim was to characterise profiles within the patient group, but were included in this post-clustering ANOVA to establish how the cluster groups differed from controls.

One-way ANOVA and Chi-squared tests were performed across cluster groups to examine any differences on demographic, symptom, diagnosis, and functioning measures. Any variables for which significant group differences were found were included as covariates in subsequent analyses. One-way ANOVAs or ANCOVAs were used to compare cluster groups across neuropsychological variables controlling for relevant covariates.

## Results

### Comparing Patients and Controls on Demographic, Sleep and Circadian Variables

Patients and controls did not significantly differ on gender distribution (χ^2^ (1) = 1.75, p = .19), but did differ in age (t(219) = 8.30, p<.001), such that controls (mean age±SD: 24.9±2.9) were significantly older than patients (mean age±SD: 21.1±3.4). Actigraphy data did not converge with the extended cosinor model for five patients and two controls, suggesting abnormal circadian rhythmicity. These individuals were removed from subsequent circadian analyses.

Compared to the control group (and controlling for age), the patient group had significantly later sleep offset times (F(1,218) = 18.00, p<.001), longer total sleep times (F(1,218) = 8.89, p<.01), increased WASO (F(1,218) = 41.59, p <.001), later sleep midpoint (F(1,218) = 6.14, p = .01), later acrophase (F(1,191) = 10.79, p<.01), and reduced R^2^ (F(1,194) = 5.11, p = .03). Sleep onset time and amplitude did not significantly differ between groups (both p >. 05).

### Assessing Relationships between Neuropsychological Variables and Sleep and Circadian Variables in Patients

Mean z scores (±SD) for neuropsychological variables ranged from 0.20±1.2 (Rey Auditory Verbal Learning Test immediate recall) to -0.62±1.3 (Rey-Osterreith Complex Figure Test 3 minute recall). Mean z scores were also low on the CANTAB Rapid Visual Information Processing (mean z score±SD: -0.47±.1.3) and Trail Making Test B (mean z score±SD:-.25±1.3). No significant correlations were found between any neuropsychological variables and sleep or circadian variables in the patient group (all p >.01; see Table 1).

**Table 1:** Correlation matrix of sleep and circadian variables with neuropsychological variables in patients.

	Sleep Onset	Sleep Offset	TST	WASO	Sleep Mid	Cosinor Acroph	Cosinor Amp	Cosinor R^2^
Trail Making Test A	0.05	0.06	0.01	0.03	0.06	0.07	0.00	-0.08
Trail Making Test B	0.12	0.12	-0.07	0.15	0.13	0.11	0.00	-0.05
RCFT- 3 min recall	0.01	0.03	-0.05	0.18	0.02	-0.01	0.11	0.10
RAVLT- immediate recall	0.04	-0.04	-0.10	-0.08	0.00	0.01	0.03	0.01
RAVLT- 20 min delayed recall	0.10	0.02	-0.16	0.08	0.06	0.03	0.07	-0.01
COWAT-letters	0.14	0.07	-0.12	0.04	0.11	0.11	-0.06	-0.16
CANTAB- 5 choice reaction time	0.12	0.13	-0.04	0.13	0.13	0.06	-0.08	-0.09
CANTAB- IED shift	-0.03	0.00	0.05	-0.06	-0.02	0.00	-0.04	-0.10
CANTAB- RVP	0.05	-0.01	-0.13	0.11	0.02	0.01	0.02	-0.06
CANTAB- Paired Associate Learning	0.00	0.02	0.02	0.01	0.01	0.00	0.03	-0.02
CANTAB- Spatial Span Length	0.00	0.04	0.02	0.07	0.02	-0.03	0.06	-0.08

TST = total sleep time; WASO = wake after sleep onset; Sleep Mid = sleep midpoint; Acroph = acrophase; Amp = amplitude; RCFT = Rey-Osterreith Complex Figure Test; RAVLT = Rey Auditory Verbal Learning Test; COWAT = Controlled Oral Word Association Test; CANTAB = Cambridge Automated Neuropsychological Testing Battery; IED = Intra/Extra Dimensional Shift; RVP = Rapid Visual Information

### Cluster Analysis using Sleep Variables

Examination of the agglomeration coefficients and dendrogram suggested a demarcation point between the three- and four-cluster solutions and an optimum solution of three clusters. One cluster group was defined by late sleep offset and long total sleep time relative to controls (‘long sleep cluster’), the second was defined by increased wake after sleep onset relative to controls (‘disrupted sleep cluster’), and the third was defined by late sleep onset and offset, and increased wake after sleep onset relative to controls (‘delayed and disrupted sleep cluster’). No significant differences were found between cluster groups on age, gender, primary diagnosis, illness duration, predicted IQ, years of education, HDRS, or SOFAS (see Table 2). As shown in Table 3 significant differences were found across cluster groups for the number of patients medication free (a greater proportion in the ‘delayed and disrupted cluster’), number of patients taking antipsychotics (a greater proportion in the ‘long sleep cluster’ and a smaller proportion in the ‘delayed and disrupted cluster’), and number of patients taking melatonin/agomelatine (a greater proportion in the ‘disrupted sleep cluster’).

**Table 2:** Mean values (± standard deviation) for sleep and demographic variables across sleep cluster groups and controls.

	1. Long Sleep Cluster (N = 31)	2. Disrupted Sleep Cluster (N = 85)	3. Delayed and Disrupted Sleep Cluster (N = 36)	Controls (N = 69)	ANOVA / x^2^	p value	significant pairwise comparisons
Sleep Onset	23.5 ± 1.3	23.6 ± 0.9	26.0 ± 1.1	24.0 ± 1.2	F (3,217) = 48.24	<.001	1,2,Ctl vs 3
Sleep Offset	9.4 ± 1.5	8.3 ± 1.0	10.7 ± 1.4	8.2 ± 1.1	F (3,81.5) = 36.93^a^	<.001	2,Ctl vs 1 vs 3
TST	536.5 ± 53.0	441.8 ± 47.1	441.1 ± 46.7	439.9 ± 45.3	F (3,217) = 36.18	<.001	2,3,Ctl vs 1
WASO	53.1 ± 16.3	81.4 ± 24.5	80.6 ± 33.0	52.6 ± 17.8	F (3,89.6) = 30.42^a^	<.001	1, Ctl vs 2,3
Age	21.0 ± 3.7	21.0 ± 3.6	21.3 ± 2.4	-	F (2,71.0) = 0.28^a^	.76	-
Gender (F/M)	24/7	57/28	19/17	-	χ^2^ (2,N = 152) = 4.63	.10	-
Primary Diagnosis (D/B/A)	15/14/2	51/25/9	21/7/8	-	χ^2^ (4,N = 152) = 8.11	.09	-
Illness Duration (years)	5.8 ± 3.1	6.6 ± 3.9	6.4 ± 3.2	-	F (2,105) = 0.38	.68	-
Predicted IQ	102.7 ± 9.8	104.2 ± 8.3	103.4 ± 8.5	-	F (2,147) = 0.34	.71	-
Years of Education	12.1 ± 2.1	12.2 ± 2.1	12.6 ± 1.7	-	F (2,145) = 0.57	.57	-
HDRS	12.1 ± 6.6	11.9 ± 7.0	13.2 ± 5.4	-	F (2,134) = 0.45	.64	-
SOFAS	63.2 ± 13.2	64.5 ± 11.1	60.6 ± 9.4	-	F (2,138) = 1.41	.25	-

Sleep onset and offset are time values in decimal form; TST = total sleep time (minutes); WASO = wake after sleep onset (minutes); Ctl = controls; ANOVA = analysis of variance; (D/B/A) = depression/anxiety/bipolar; HDRS = Hamilton Depression Rating Scale (not including insomnia items); SOFAS = Social and Occupational Functioning Scale

a- Welch statistic used due to significantly unequal variance between groups

**Table 3:** Medication counts and percentages across sleep cluster groups.

Medication		1. Long Sleep Cluster (N = 31)	2. Disrupted Sleep Cluster (N = 85)	3. Delayed and Disrupted Sleep Cluster (N = 36)	χ^2^	p value
None	Count	4	19	14	χ^2^ (2,N = 138) = 6.73	<.05
	%	13.8%	25.0%	42.4%		
Antidepressant	Count	15	36	13	χ^2^ (2,N = 138) = 1.01	.60
	%	51.7%	47.4%	39.4%		
Antipsychotic	Count	15	22	5	χ^2^ (2,N = 138) = 9.93	<.01
	%	51.7%	28.9%	15.2%		
Mood Stabilizers	Count	10	19	4	χ^2^ (2,N = 138) = 4.35	.11
	%	34.5%	25.0%	12.1%		
Sedatives/Hypnotics	Count	3	5	5	χ^2^ (2,N = 138) = 2.02	.37
	%	10.3%	6.6%	15.2%		
Melatonin/Agomelatine	Count	1	13	1	χ^2^ (2,N = 138) = 6.79	<.05
	%	3.4%	17.1%	3.0%		
Other	Count	1	2	0	χ^2^ (2,N = 138) = 1.03	.60
	%	3.4%	2.6%	0%		

**Fig 1:**
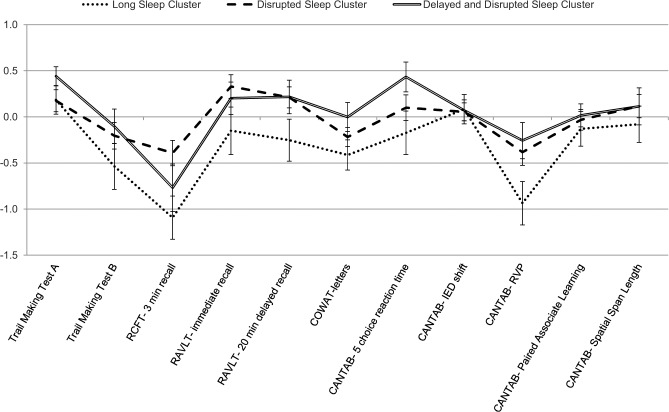
Profile of neuropsychological performance across sleep cluster groups. Error bars represent standard error. RCFT = Rey-Osterreith Complex Figure Test; RAVLT = Rey Auditory Verbal Learning Test; COWAT = Controlled Oral Word Association Test; CANTAB = Cambridge Automated Neuropsychological Testing Battery; IED = Intra/Extra Dimensional Shift; RVP = Rapid Visual Information Processing.

Fig 1 shows z-scores for neuropsychological performance across sleep cluster groups. A main effect of cluster group was found for the Rey-Osterreith Complex Figure Test 3 minute recall scores such that the long sleep cluster performed significantly worse than the disrupted sleep cluster (F(2,144) = 3.29, p = .04). There were no significant differences on any other neuropsychological variables (all p >.05).

### Cluster Analysis using Circadian Variables

Examination of the agglomeration coefficients and dendrogram suggested a demarcation point between the three- and four-cluster solutions and an optimum solution of three clusters. One cluster group was defined by higher amplitude and R^2^ relative to controls (‘strong circadian cluster’), the second was defined by lower amplitude and R^2^ relative to controls (‘weak circadian cluster’), and the third was defined by moderately lower R^2^ and delayed acrophase and sleep midpoint relative to controls (‘delayed circadian cluster’). No significant differences were found between cluster groups on gender, primary diagnosis, predicted IQ, HDRS, or SOFAS. However, a significant difference was found between the strong circadian cluster and the weak circadian cluster on age and illness duration, such that the strong circadian cluster were younger and had a shorter illness duration (see Table 4). As shown in Table 5, significant differences were found across cluster groups for the number of patients medication free (a greater proportion in the delayed circadian cluster), and number of patients taking antipsychotics (a smaller proportion in the delayed circadian cluster).

**Table 4:** Mean values (± standard deviation) for circadian and demographic variables across circadian cluster groups and controls.

	1. Strong Circadian Cluster (N = 76)	2. Weak Circadian Cluster (N = 26)	3. Delayed Circadian Cluster (N = 31)	Controls (N = 67)	ANOVA / χ^2^	p value	significant pairwise comparisons
Sleep Midpoint	3.9 ± 0.9	4.6 ± 1.0	6.2 ± 1.2	4.1 ± 1.0	F(3,198) = 45.22	<.001	1,2,Ctl vs 3; 1 vs 2
Acrophase	15.6 ± 0.9	16.1 ± 1.0	18.0 ± 1.1	15.6 ± 0.9	F(3,187) = 47.80	<.001	1,2,Ctl vs 3
Amplitude	2.0 ± 0.36	1.26 ± 0.24	1.75 ± 0.24	1.65 ± 0.31	F(3,188) = 36.40	<.001	3,Ctl vs 1 vs 2
R^2^	0.48 ± 0.08	0.24 ± 0.07	0.37 ± 0.10	0.42 ± 0.10	F(3,188) = 50.15	<.001	Ctl vs 1 vs 2 vs 3
Age	20.2 ± 3.3	23.0 ± 3.0	21.0 ± 3.0	-	F(2,130) = 7.13	<.01	1 vs 2
Gender (F/M)	53/23	16/10	20/11	-	χ^2^ (2,N = 133) = 0.69	.71	
Primary Diagnosis (D/B/A)	46/20/10	15/11/0	19/7/5	-	χ^2^ (2,N = 133) = 6.09	.19	
Illness Duration (years)	5.6 ± 3.5	7.8 ± 3.5	5.5 ± 2.9	-	F(2,91) = 3.84	<.05	1 vs 2
Predicted IQ	103.1 ± 9.7	105.6 ± 6.8	103.3 ± 7.8	-	F(2,128) = 0.81	.45	
Years of Education	11.8 ± 2.0	13.0 ± 2.1	12.2 ± 1.9	-	F(2,126) = 2.97	.06	
HDRS	11.1 ± 6.6	14.1 ± 7.1	11.7 ± 4.5	-	F(2,116) = 2.06	.13	
SOFAS	64.1 ± 10.8	61.8 ± 14.1	60.2 ± 11.3	-	F(2,120) = 1.19	.31	

Sleep midpoint is time values in decimal form; Ctl = controls; ANOVA = analysis of variance; (D/B/A) = depression/anxiety/bipolar; HDRS = Hamilton Depression Rating Scale (not including insomnia items); SOFAS = Social and Occupational Functioning Scale

**Table 5:** Medication counts and percentages across circadian cluster groups.

Medication		1. Strong Circadian Cluster (N = 76)	2. Weak Circadian Cluster (N = 26)	3. Delayed Circadian Cluster (N = 31)	χ^2^	p value
None	Count	14	4	13	χ^2^ (2,N = 120) = 9.12	<.05
	%	20%	17.4%	48.1%		
Antidepressant	Count	36	12	9	χ^2^ (2,N = 120) = 2.81	.25
	%	51.4%	52.2%	33.3%		
Antipsychotic	Count	24	9	3	χ^2^ (2,N = 120) = 6.11	<.05
	%	34.3%	39.1%	11.1%		
Mood Stabilizers	Count	16	8	4	χ^2^ (2,N = 120) = 2.79	.25
	%	22.9%	34.8%	14.8%		
Sedatives/Hypnotics	Count	7	3	1	χ^2^ (2,N = 120) = 1.44	.49
	%	10%	13%	3.7%		
Melatonin/Agomelatine	Count	11	4	0	χ^2^ (2,N = 120) = 5.02	.08
	%	15.7%	17.4%	0.0%		
Other	Count	2	0	0	χ^2^ (2,N = 120) = 1.45	.48
	%	2.9%	0%	0%		

**Fig 2:**
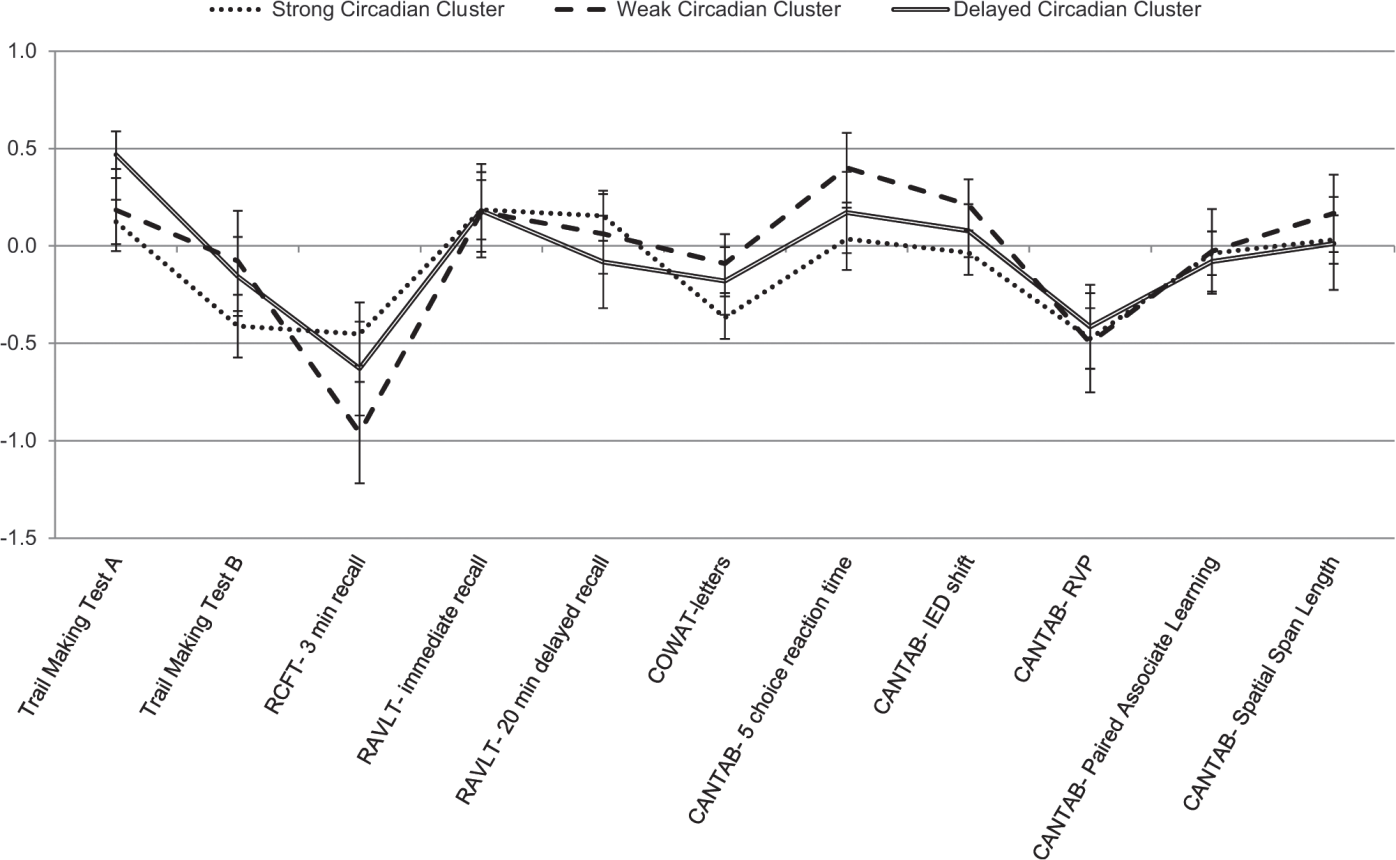
Profile of neuropsychological performance across circadian cluster groups. Error bars represent standard error. RCFT = Rey-Osterreith Complex Figure Test; RAVLT = Rey Auditory Verbal Learning Test; COWAT = Controlled Oral Word Association Test; CANTAB = Cambridge Automated Neuropsychological Testing Battery; IED = Intra/Extra Dimensional Shift; RVP = Rapid Visual Information Processing

Fig 2 shows z-scores for neuropsychological performance across circadian cluster groups. No significant differences were found between the circadian cluster groups on any neuropsychological variables (all p >.05).

## Discussion

This study examined the relationship between sleep, circadian rhythms, and cognition in young people with affective disorders. Patients presented with a profile of abnormal sleep and circadian function characterised by poor sleep consolidation, prolonged sleep periods, late wake times, late activity peak and reduced strength of the rest-activity rhythm. This is largely in agreement with abnormalities reported in previous studies in patients with affective disorders [18, 19, 22, 28, 32–34, 87]. Although an overall delay of sleep onset [33], and reduced circadian amplitude [36] were not found in the current sample, subgroups of patients presented with these characteristics. Overall neuropsychological performance in patients was in the average to low average range, with poorest scores on tasks measuring visual memory and mental flexibility. Correlations between neuropsychological performance and sleep or circadian measures found no simple linear relationships between these variables.

We identified three distinct patterns of sleep in our sample. One was a ‘disrupted sleep cluster’ which showed poor sleep consolidation reflected by increased WASO relative to controls. A ‘delayed and disrupted sleep cluster’ showed similar sleep consolidation deficits (increased WASO), accompanied by a delay in sleep phase, with later sleep onset and offset times. Finally, a ‘long sleep cluster’ was no different to controls on WASO or sleep onset, but was found to have a delayed sleep offset time and longer total sleep time. The long sleep cluster had a greater proportion of participants taking antipsychotics, which is likely to contribute to the increased sleep time in this group [88]. The delayed and disrupted cluster had a smaller proportion of participants taking antipsychotics and a greater proportion taking no medication. Patients in the disrupted sleep cluster were more likely to be taking melatonin or agomelatine. Melatonin and its analogues exert effects on the timing of sleep, with the potential to realign abnormal sleep-wake cycles [89–91]. Consistent with this, the disrupted sleep cluster differed from controls only on WASO and did not show abnormalities in sleep timing. However, incidence of melatonin and agomelatine use was low overall and as such cannot fully explain the findings.

In contrast to our hypothesis, the long sleep cluster showed a pattern of poorer cognitive performance, with some degree of impairment across most neuropsychological measures, and significantly poorer performance on the immediate recall of the Rey Complex Figure Test (RCFT) relative to the disrupted sleep cluster. Previous research suggests that visual memory may be particularly sensitive to dysfunction in psychiatric patients [92–94], and may be linked to neurobiological changes [95, 96]. This is consistent with our finding of poorer RCFT performance despite no significant differences across other cognitive measures.

Shorter sleep length and more disturbed sleep have typically been associated with poorer cognitive performance in healthy individuals [49–52] and in older adults with depression [59–61], thus our result of poorer visual memory performance in the cluster with the longest sleep time and no significant disruption in sleep is unexpected. In direct contrast to our current findings, Goder and colleagues [62] reported previously that poorer recall performance on the RCFT was associated with a shorter total sleep time and a lower amount of REM sleep in adults with major depressive disorder. This discrepancy in findings could be due to measurement of different underlying mechanisms; Goder’s findings were related to an overnight recall period and a single polysomnographic recording, and thus a more acute effect of sleep. Additionally, the patients in Goder’s study were free of medication, whereas the long sleep cluster in the current study had an increased use of antipsychotics. Antipsychotic use has been shown to improve aspects of cognitive performance in psychotic disorders [97–99], but studies in patients with affective disorders suggest neutral [100] or negative effects on cognition [93, 101–104]. Hence, medication effects provide a possible explanation for the current findings.

An alternate explanation is that Goder’s findings in relation to REM sleep may speak to a fundamental disruption in the neurobiological process of sleep in affective disorders, which may exist in both abnormally short and abnormally long sleep length and could lead to poorer cognitive functioning. As none of our cluster groups differed across diagnostic categories or symptom measures, this link between visual memory and longer sleep appears to be independent of mechanisms influencing affective symptoms. Various aspects of brain activity during sleep have been related to cognitive performance in healthy children and adults [105–108] and a second study by Goder and colleagues [63] reports that reduced SWS is correlated with poorer verbal memory retention in a moderately depressed sample. Additionally, evidence from brain imaging studies highlights a potential role of dysfunctional neural networks in affective disorder, which may contribute to both sleep and cognitive alterations [109]. Hence, in addition to the potential effects of antipsychotic intake in several patients from the long sleep cluster, abnormal brain functioning during sleep may be an underlying cause of the current findings of poorer visual memory function in the long sleep cluster despite a presumably adequate amount of sleep. In addition to driving disruptions in cognitive function, abnormal brain function during sleep in these individuals may provide inadequate restorative effects of sleep, resulting in increased tiredness and extension of the sleep episode as a compensatory effect. In concord with this, abnormalities in sleep EEG are reported in bipolar and major depressive disorders, including disturbances in REM and SWS [22, 110, 111], and hypersomnia in major depression has been associated with reduced SWS [112].

Affective disorders have a considerable amount of heterogeneity in symptoms and trajectories [113], as do sleep and circadian rhythm disturbances [65]. Hence, a particular profile of sleep and cognition may be reflective of a variety of contributing factors. Interestingly, none of our cluster groups differed in terms of symptoms or diagnosis, although they did differ in medication. This may reflect certain profiles of sleep dysfunction as general risk factors for multiple disorders, or common aspects of different disorders causing various sleep abnormalities. It also illustrates the potential utility of profiling based on other measures of symptoms, functioning, or related factors, beyond traditional diagnostic categories. Such profiling may ultimately provide a better understanding of mental illness in youth and may even help to predict treatment response, in turn informing more targeted personalisation interventions.

Three distinct patterns of circadian function were also identified in our sample. One ‘delayed circadian cluster’ showed a delayed circadian phase, with delayed sleep midpoint and peak of activity, as well as slightly reduced strength of the circadian rhythm of the rest-activity cycle. The other two cluster groups had no difference in the timing of circadian phase relative to controls. The ‘strong circadian cluster’ showed increased amplitude and strength of the circadian activity rhythm, and the ‘weak circadian cluster’ showed decreased amplitude and strength of the circadian activity rhythm. The difference between the strong and weak circadian clusters may be explained in part by developmental changes in circadian rhythmicity [87, 114], as the strong circadian cluster were significantly younger than the weak circadian cluster. The delayed circadian cluster had a greater proportion of patients taking no medication, and a smaller proportion taking antipsychotics. As with the sleep clusters, the circadian clusters did not differ in terms of symptoms or diagnosis.

The three circadian cluster groups did not differ in terms of cognitive performance. This is in contrast to our hypothesis and to previous findings of associations between circadian disruption and cognition [53–55]. Our results suggest that particular profiles of circadian rhythms do exist in affective patients, but these may not be directly related to cognitive performance.

Our study is limited by its cross sectional design which does not allow us to determine directions of causality. In addition, potential selection bias due to the help-seeking nature of participants may limit the generalizability of the findings. Medication differed between clusters and thus effects of medication cannot be easily separated from other aspects of affective illness. Another potential confound is time of day of testing, which has been shown to affect neuropsychological performance [58, 115] and would likely interact with individual sleep and circadian profiles. This study would also have benefited from a control group with neuropsychological data to further clarify cognitive dysfunction in different cluster groups, although it is noted that we used standardised scores based on appropriate normative data.

To our knowledge, this is the first study to report on relationships between cognition and sleep and circadian profiles in young people with affective disorders. It is also unique in the use of a cluster analysis technique to define groups of such patients based on sleep and circadian rhythms. Replication in further samples of young people is necessary to validate our cluster solutions and increase the generalizability of the findings. Future research should also use polysomnographic recordings and measurement of brain function during wake to elucidate the underlying mechanisms of sleep, circadian, and cognitive dysfunction and more precisely define how the three are interrelated in young affective disordered populations. The specific effects of different medications as distinct from other aspects of affective disorders require more detailed investigation. Longitudinal studies are also required to establish directions of causality.

In conclusion, we found distinct profiles of sleep and circadian rhythms in young persons with affective disorders. Sleep and circadian rhythms did not have simple linear relationships with cognitive performance in young persons with affective disorders; however, a cluster of patients defined by longer sleep time and later wake up time, half of whom were taking antipsychotic medication, had significantly worse performance on a visual memory task. Our results are partially supportive of a relationship between sleep and cognition in young people with affective disorders, although the specific mechanisms of this relationship remain to be established. Exploration of these mechanisms may highlight potential treatment targets (i.e. abnormal brain functioning) that may result in greater effectiveness of treatment and facilitate improvements across domains of cognition, sleep, and circadian functioning as well as reducing affective symptoms. Our study also demonstrates distinct profiles of sleep and circadian rhythms in young patients which were not linked to specific diagnostic categories or levels of symptom severity, highlighting the heterogeneity of sleep and circadian factors in these patients. This has implications in terms of targeted interventions for sleep and circadian function, as individuals with specific sleep and circadian profiles may differ in their need and responsiveness to such treatment. Thus, profiling based on sleep and circadian measures has potential utility in the personalisation of treatments.

## Supporting Information

S1 Dataset Raw data file. (XLSX)
